# An improved methodology for quantifying causality in complex ecological systems

**DOI:** 10.1371/journal.pone.0208078

**Published:** 2019-01-25

**Authors:** Hiroko Kato Solvang, Sam Subbey

**Affiliations:** 1 Marine Mammals Research Group, Institute of Marine Research, Bergen, Norway; 2 Research Group on Fisheries Dynamics, Institute of Marine Research, Bergen, Norway; 3 Department of Natural Resources, Cornell University, Ithaca, New York, United States of America; Northwestern University, UNITED STATES

## Abstract

This paper provides a statistical methodology for quantifying causality in complex dynamical systems, based on analysis of multidimensional time series data of the state variables. The methodology integrates Granger’s causality analysis based on the log-likelihood function expansion (Partial pair-wise causality), and Akaike’s power contribution approach over the whole frequency domain (Total causality). The proposed methodology addresses a major drawback of existing methodologies namely, their inability to use time series observation of state variables to quantify causality in complex systems. We first perform a simulation study to verify the efficacy of the methodology using data generated by several multivariate autoregressive processes, and its sensitivity to data sample size. We demonstrate application of the methodology to real data by deriving inter-species relationships that define key food web drivers of the Barents Sea ecosystem. Our results show that the proposed methodology is a useful tool in early stage causality analysis of complex feedback systems.

## Introduction

The first attempt at deriving causal inference between variables goes back to a study on feedback systems by Wiener [1], where by his definition, a given time series is *causal* to another if knowledge of the first series reduces the mean square prediction error of the second. Granger [2] followed this notion of causality, and applied it to the analysis of economic time series data. The goal was to explore the causal relationship between two variables that were selected from what was considered as a multidimensional complex feedback system. Granger applied bivariate time series models within the time domain, and based on this, defined the prediction error as a metric for assessing model results. Geweke [3] expanded on Granger’s idea to define a measure for model comparison based on using the quasi-likelihood function. A parallel development to Granger’s approach is by Akaike [4], who provided a feedback system analysis based on a multivariate auto-regressive model. Here, we define feedback as being present when given bivariate time series, each of them is mutually causal to the other [5]. Akaike’s approach was a practical statistical method to investigate mutual relationship among variables from two different angles—the open/closed impulse response calculated in time domain, and the relative power contribution calculated in frequency domain. The methodology is referred to as the Akaike’s total causality approach**.** Numerous successful applications of the method can be found in several fields such as engineering, physics, economics and medical science [6].

A similar approach to the relative power contribution using multivariate auto-regressive models, the directed coherence method, has been introduced by Baccalá and Sameshima [7]. The directed coherence method however, focused mainly on the use of auto-regressive coefficients to capture the transfer characteristics and ignored the noise contribution to the system. Noise however, is an important information for dynamical system evolution, and is referred to as the *innovation* in dynamic system modeling [5]. Thus auto-regressive coefficients and noise contribution both play important roles in the Akaike causality, as well as in the Granger causality formulations [8].

In the summary on causality analysis presented in Section 14 of [5], the following two concepts are integrated—the Granger and Geweke type pair-wise causality, and the Akaike’s total causality. The Granger-Geweke pair-wise causality can be derived for each pair of the variables in a multidimensional (over two-dimensional) feedback system from the Akaike’s total causality of the system. It must be noted that while it is possible to derive pair-wise causality from assessment of total causality, the opposite process (i.e., the integration of multiple pair-wise causality measures to obtain the total causality information of a system) is non-trivial. The identification of total causality is therefore the most important analysis procedure in the study of complex multivariate feedback systems [5].

Despite novelty of the idea, using Akaike total causality to assess Granger-Geweke type pair-wise causality information has hitherto not been applied to complex systems, where such information is central for understanding and prediction of system dynamics.

The goal of this paper is to provide a new numerical algorithm for the integrated causality approach, and to demonstrate its efficacy through application to simulated data, and to empirical observations. The simulation data is generated by several auto-regressive process models. The performance of the method is evaluated with respect to sensitivity of the causal inference to the sample size of the observation data. Based on results from the simulation study, we apply the methodology to investigate inter-species and environmental factors that act as causal drivers of the capelin fish stock in the Barents Sea.

The Barents Sea, like many marine ecosystems, is characterized by several levels of interactions between marine species (populations of fish, birds, sea mammals etc.) and the marine environment [9]. Understanding the nature of these interactions is imperative because they define ecosystem functioning, and provide insight into the effect of e.g., climatic change, on the marine system dynamics [10]. Information about the interaction between marine species and the environment often exists in the form of time series data obtained from scientific surveys (see e.g. [11] and [12]). The time series data may be obtained from direct measurements (e.g., temperature) or from processed information (e.g., conversion of acoustic observations to population indices of abundance). Information about ecosystem state and function is therefore usually derived from collation of data spanning different temporal and spatial scales of observation. The goal is to demonstrate the efficacy of our methodology for causal inference by applying it to multidimensional temporal data from the Barents Sea.

### Method

Our causality analysis method involves two procedures namely, application of a Multivariate Autoregressive (MAR) model to capture the inter-component relationships, and a frequency domain analysis using the estimated MAR coefficients and the covariance matrix of the prediction error. The frequency domain analysis is an important procedure for physical interpretation of the estimated MAR model parameters. We use the Akaike noise contribution approach to quantify the pair-wise causal link between variables. We show that unlike other pair-wise causality methodologies (e.g., the Granger-Geweke’s approach) our algorithm only requires the application of a bi-variate model in assessing the presence or absence of a causal relationship. In the sections to follow, we introduce: 1. the MAR model and the model selection procedure, 2. the Akaike noise contribution approach, 3. the Granger-Geweke type pair-wise causality analysis, and finally 4. A causal inference method metric- delta log-likelihood values—that is based on integrating the pair-wise causal inference and Akaike’s total power contribution.

#### The multivariate auto-regressive model and model selection

Let the observed *k*-dimensional time series be denoted by **x**_*t*_ = (x_1_(*t*), x_2_(*t*), ⋯, x_*k*_ (*t*))*'* (*t* = 1,⋯, *N*), where (⋅)' denotes transposition. We assume that the variables are mutually interactive in a system that is driven by a multivariate auto-regressive (MAR) process

$${\text{x}}_{t}=\sum_{m=1}^{M}A_{m}x_{t-m}+\varepsilon_{t},$$ (1)

where *M* is the AR order, **A**_*m*_ is the AR coefficients matrix, and **ε**_*t*_ follows a multinomial distribution with mean zero vector and variance-covariance matrix Σ. The AR coefficients matrix can be estimated by the Ordinary Least Squares (OLS) method. Other numerical algorithm such as the Yule-Walker or alternatively, the Levinson’s method, can be applied to obtain stable estimates [6]. The AR order is identified by statistical model selection approaches, such as, the Akaike Information Criterion (AIC) [2], defined by

AIC=−2×log−likelihood+2×#model’sparameters. (2)

#### Akaike noise contribution

The physical interpretation of the estimated AR coefficients is obtained by considering the following procedure in the frequency domain. First, the cross-power spectra **P**_*f*_ of the data is given by

$$P_{f}=F_{f}\Sigma F_{f}^{*}=\left(\begin{array}{cccc} p_{11f} & p_{12f} & \cdots & p_{1kf}\\ p_{21f} & p_{22f} & \cdots & p_{2kf}\\ & \vdots & & \\ p_{k1f} & p_{k2f} & \cdots & p_{kkf} \end{array}\right),0\leq f\leq 0.5\Delta ,$$ (3)

where Δ indicates a sampling interval, and $F_{f}^{*}$ is the complex conjugate of **F**_*f*_, which is the frequency response defined by

$$F_{f}={\left\{I-\sum_{m=1}^{M}A_{m}e^{-2\pi fm}\right\}}^{-1}.$$ (4)

**I** is the identity matrix, and the off-diagonal components of **P**_*f*_ represent the cross-power spectrum. Next, assuming Σ is of diagonal form, the power spectrum of *x*_*i*_ *is* formed as sum of terms by the *j*^th^ frequency response function *F*_*ij f*_ and the variance $\sigma_{jj}^{2}$ for the prediction error of *x*_*j*_ by

$$p_{ii\;f}={\left|F_{11\;f}\right|}^{2}\sigma_{11}^{2}+{\left|F_{12\;f}\right|}^{2}\sigma_{22}^{2}+\cdot \cdot \cdot +{\left|F_{1k\;f}\right|}^{2}\sigma_{kk}^{2}.$$ (5)

The effect from the noise of *j*^th^ variable *x*_*j*_ is given by

$$r_{ij\;f}=\frac{{\left|F_{ij\;f}\right|}^{2}\sigma_{jj}^{2}}{\left|p_{ii\;f}\right|}\in \left[0,\;1\right],$$ (6)

which is called the *Relative Power contribution* (*RPC*) or the Akaike’s RPC, after its originator [4]**.** The extended power contribution approach, introduced by Tanokura and Kitagawa [13], deals with situations involving significant correlated noise. Following [5], since (5) integrates causality relationships among all variables, it is called the *total causality* of the whole variable set and (6) is referred to as the *partial innovation contribution* ratio. The computed value *r*_*ij f*_ is called the *partial causality*.

#### Granger causality

Here, we briefly review the basic concept for Granger causality [14]. For the observed two-dimensional time series data *x*_1*t*_ and *x*_2*t*_, we consider a time series model, with a variance of prediction error Var(*x*_1,*t*_ | *U*_*s*_), where *U*_*s*_ is the universal set that contains all information about the system being modeled, up to time *s*. Granger causality is defined as

Definition1.

If Var(*x*_1,t_ | *U*_*t*−*l*_) < Var(*x*_1*t*_ | *U*_*t*−*l*_ − *x*_2,*t*−*l*_), *x*_2,*t*_ causes *x*_1,*t*_.

Definition2.

If Var(*x*_1,t_ | *U*_*t*−*l*_) < Var(*x*_1*t*_ | *U*_*t*−*l*_ − *x*_2,*t*−*l*_) and Var(*x*_2,t_ | *U*_*t*−*l*_) < Var(*x*_2,*t*_ | *U*_*t*−*l*_ − *x*_1,*t*−*l*_), since *x*_1*t*_ also causes *x*_2*t*_ a feedback relation exists between *x*_1*t*_ and *x*_2*t*_.

Geweke [15] generalized Granger’s *Definition 1* as:

Definition3.

The time series vector *x*_1*t*_ Geweke-causes another time series *x*_2*t*_ if log | Var(*x*_1*t*_ | *x*_1*t*−_)|− log | Var(*x*_1*t*_ | *x*_1*t*−_,*x*_2*t−*_) | > 0, where *x*_1,*t*−_ = (*x*_1,*t*−1_,*x*_1,*t*−2_,⋯)' and *x*_2,*t*−_ = (*x*_2,*t*−1_,*x*_2,*t*−2_,⋯)'.

Here, Var(*x*_1*t*_ | *x*_1*t*−_) is the variance of the prediction error of *x*_1,*t*_ obtained by Model^(0)^, which is a prediction model for *x*_1,*t*_ with only *x*_1,*t*−_. On the other hand, Var(*x*_1,*t*_ | *x*_1,*t*−_,*x*_2,*t*−_) is the variance of the prediction error of *x*_1,*t*_, obtained by Model^(1)^, which is a prediction model for *x*_1,*t*_ with both *x*_1,*t*−_ and *x*_2,*t*−_. Ozaki [5] points out that the mathematical criterion of Granger and Geweke are essentially equivalent to the following general criterion, given by Definition 4:

Definition4.

The observed *x*_2,*t*_ causes another observed *x*_1,*t*_, in the sense of ‘log-likelihood’, when

$$-2\text{log}p^{{\text{Model}}^{\left(0\right)}}(x_{1\;t})-2\text{log}p^{{\text{Model}}^{\left(1\right)}}(x_{1\;t})\;>0.$$

The pairwise total causality assesses the difference in likelihood between the model with, and without causal influence from other variables.

#### Pair-wise causal inference based on Akaike’s total power contribution

Kolmogorov [16] defined a relationship between the variance of the prediction error *σ*^2^ of a stationary process and the power spectrum *p*(*f*) by:

$$\text{log}\sigma^{2}=\int_{-1/2}^{1/2}\text{log}p(f)df.$$ (7)

Using (7), the power spectrum *p*_*ii*,*f*_ in (3) can be expressed in terms of log value of the variance of the prediction error for the *i*^th^ variable. We derive

$$\begin{array}{ll} \text{log}\sigma_{i}^{2}=\int_{-1/2}^{1/2}\text{log}p_{ii}(f)df & =\int_{-1/2}^{1/2}\text{log}\sum_{k=1}^{K}{\left|\alpha_{ik}(f)\right|}^{2}\sigma_{k}^{2}df\\ & =\int_{-1/2}^{1/2}\text{log}({\left|F_{i1}(f)\right|}^{2}\sigma_{1}^{2}+{\left|F_{i2}(f)\right|}^{2}\sigma_{2}^{2}+\cdot \cdot \cdot +{\left|F_{iK}(f)\right|}^{2}\sigma_{K}^{2})df. \end{array}$$ (8)

In the original MAR model, if we exclude the contribution of the noise effect from the *j*^th^ to the *i*^th^ variable, the logarithm of the variance for the prediction error, logσi^j2, is given by

logσi^j2=∫−1/21/2logpii(j)(f)df=∫−1/21/2log(∑k=1j−1|αik(f)|2σk2+∑k=j+1k|αik(f)|2σk2)df. (9)

The difference between logσi^j2 and $\text{log}\sigma_{i}^{2}$ (see [15]) can be written as

logσi^j2−logσii2=∫−1/21/2logpii(j)(f)df−∫−1/21/2logpii(f)df=∫−1/21/2logpii(j)(f)pii(f)df=∫−1/21/2logpii(f)−|αij(f)|σjj2pii(f)df=∫−1/21/2log(1−|αij(f)|σjj2pii(f))df≈∫−1/21/2|αij(f)|σjj2pii(f)df=∫−1/21/2rij,fdf, (10)

which is equivalent to the integration of the noise contribution from the *j*^th^ to the *i*^th^ variable in the whole frequency domain. This measure defines the Granger-Geweke type pair-wise causality that is derivable from the Akaike’s total causality relationship [5]. In the rest of this manuscript, ΔLL will represent the difference between log-likelihoods for two models as given by (10).

For a given value for ΔLL, it is desirable to define a threshold level for assessing the significance of the *j*^th^ variable causal contribution to the dynamic of the *i*^th^ variable. This article defines such a threshold level based on the AIC concept [17]. Using the model comparison given in (10), the difference between AIC for a full model and AIC for the model that excludes noise from the *j*^th^ variable is given by

AICσi^j2−AICσii2=−2×logσi^j2+2×(k−1)−{−2×logσii2+2×k}=ΔLL−2. (11)

**Fig 1:**
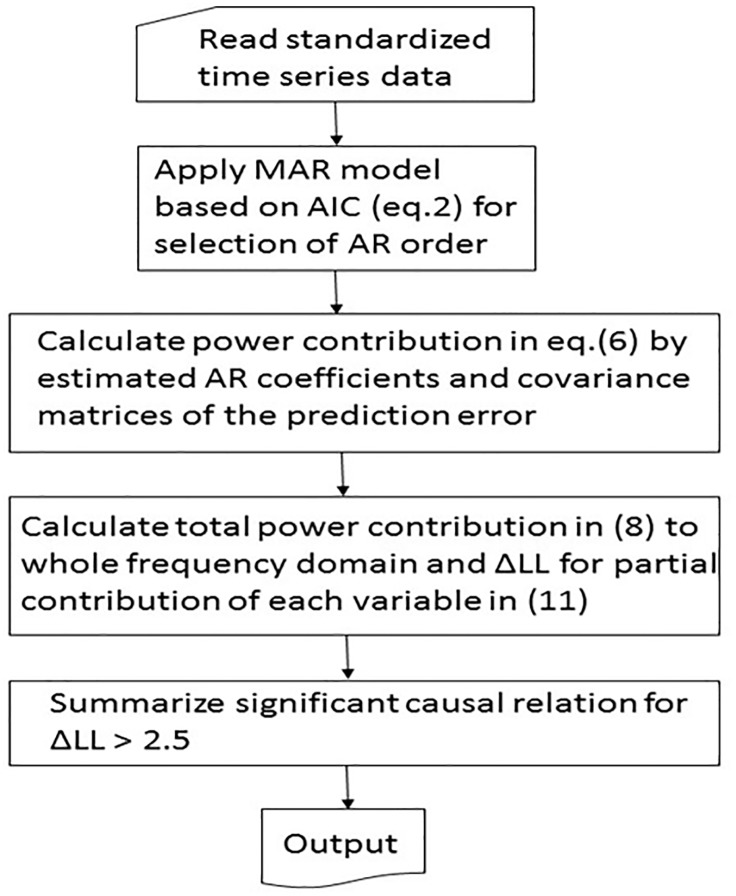
Block diagram of the algorithm summarizing the calculating procedure of ΔLL.

The implication of (11) is that, it is difficult to assume the existence of a significant influence from *j* to *i* if ΔLL < 2. We summarize the procedure in the algorithm shown in Fig 1.

### Simulation study

This paper uses numerical simulations for illustration, and in evaluating the proposed methodology. We present two types of simulation studies (labelled as Study 1 and Study 2) where, the goal of Study 1 is to confirm the accuracy of the causal inference by MAR model including full variables, and Study 2 aims at confirming the performance of the proposed ΔLL metric for several simulated multi-dimensional data.

We first generate four types of multidimensional simulation data using AR models, where the model order is fixed, for the sake of simplicity. Four datasets were generated using the following coefficients:

1. 3 variables:

$$\left(\begin{array}{ccc} a_{11} & a_{12} & a_{13}\\ a_{21} & a_{22} & a_{23}\\ a_{31} & a_{32} & a_{13} \end{array}\right)=\left(\begin{array}{ccc} 0.5 & 0.3 & \\ & 0.5 & 0.4\\ & & 0.5 \end{array}\right),$$

2. 5 variables:

$$\left(\begin{array}{ccccc} a_{11} & a_{12} & a_{13} & a_{14} & a_{15}\\ a_{21} & a_{22} & a_{23} & a_{24} & a_{25}\\ a_{31} & a_{32} & a_{13} & a_{34} & a_{35}\\ a_{41} & a_{42} & a_{43} & a_{44} & a_{45}\\ a_{51} & a_{52} & a_{53} & a_{54} & a_{55} \end{array}\right)=\left(\begin{array}{ccccc} 0.7 & 0.5 & & 0.6 & \\ & 0.7 & & & \\ & & 0.7 & & 0.6\\ & & & 0.7 & 0.6\\ & & & & 0.7 \end{array}\right),$$

3. 8 variables case 1:

$$\left(\begin{array}{cccccccc} a_{11} & a_{12} & a_{13} & a_{14} & a_{15} & a_{16} & a_{17} & a_{18}\\ a_{21} & a_{22} & a_{23} & a_{24} & a_{25} & a_{26} & a_{27} & a_{28}\\ a_{31} & a_{32} & a_{33} & a_{34} & a_{35} & a_{36} & a_{37} & a_{38}\\ a_{41} & a_{42} & a_{43} & a_{44} & a_{45} & a_{46} & a_{47} & a_{48}\\ a_{51} & a_{52} & a_{53} & a_{54} & a_{55} & a_{56} & a_{57} & a_{58}\\ a_{61} & a_{62} & a_{63} & a_{64} & a_{65} & a_{66} & a_{67} & a_{68}\\ a_{71} & a_{72} & a_{73} & a_{74} & a_{75} & a_{76} & a_{77} & a_{78}\\ a_{81} & a_{82} & a_{83} & a_{84} & a_{85} & a_{86} & a_{87} & a_{88} \end{array}\right)=\left(\begin{array}{cccccccc} 0.5 & & & & & & & \\ 0.4 & 0.5 & & & & & & \\ 0.3 & & 0.5 & 0.3 & & & & \\ & & & 0.5 & & & & \\ & & & & 0.5 & & & \\ & & & & 0.3 & 0.5 & & \\ & & 0.4 & & 0.4 & & 0.5 & \\ & & & & & & 0.3 & 0.5 \end{array}\right),$$

and

4. 8 variable case 2:

$$\left(\begin{array}{cccccccc} a_{11} & a_{12} & a_{13} & a_{14} & a_{15} & a_{16} & a_{17} & a_{18}\\ a_{21} & a_{22} & a_{23} & a_{24} & a_{25} & a_{26} & a_{27} & a_{28}\\ a_{31} & a_{32} & a_{33} & a_{34} & a_{35} & a_{36} & a_{37} & a_{38}\\ a_{41} & a_{42} & a_{43} & a_{44} & a_{45} & a_{46} & a_{47} & a_{48}\\ a_{51} & a_{52} & a_{53} & a_{54} & a_{55} & a_{56} & a_{57} & a_{58}\\ a_{61} & a_{62} & a_{63} & a_{64} & a_{65} & a_{66} & a_{67} & a_{68}\\ a_{71} & a_{72} & a_{73} & a_{74} & a_{75} & a_{76} & a_{77} & a_{78}\\ a_{81} & a_{82} & a_{83} & a_{84} & a_{85} & a_{86} & a_{87} & a_{88} \end{array}\right)=\left(\begin{array}{cccccccc} 0.5 & 0.3 & & 0.4 & & & 0.3 & \\ & 0.5 & 0.3 & & & & & \\ & & 0.5 & & 0.4 & & & \\ & & & 0.5 & & & & \\ 0.3 & & & & 0.5 & & & \\ & & & & & 0.5 & 0.2 & 0.3\\ & 0.3 & & 0.3 & & & 0.5 & 0.3\\ & & & & & 0.3 & 0.2 & 0.5 \end{array}\right)$$

**Fig 2:**
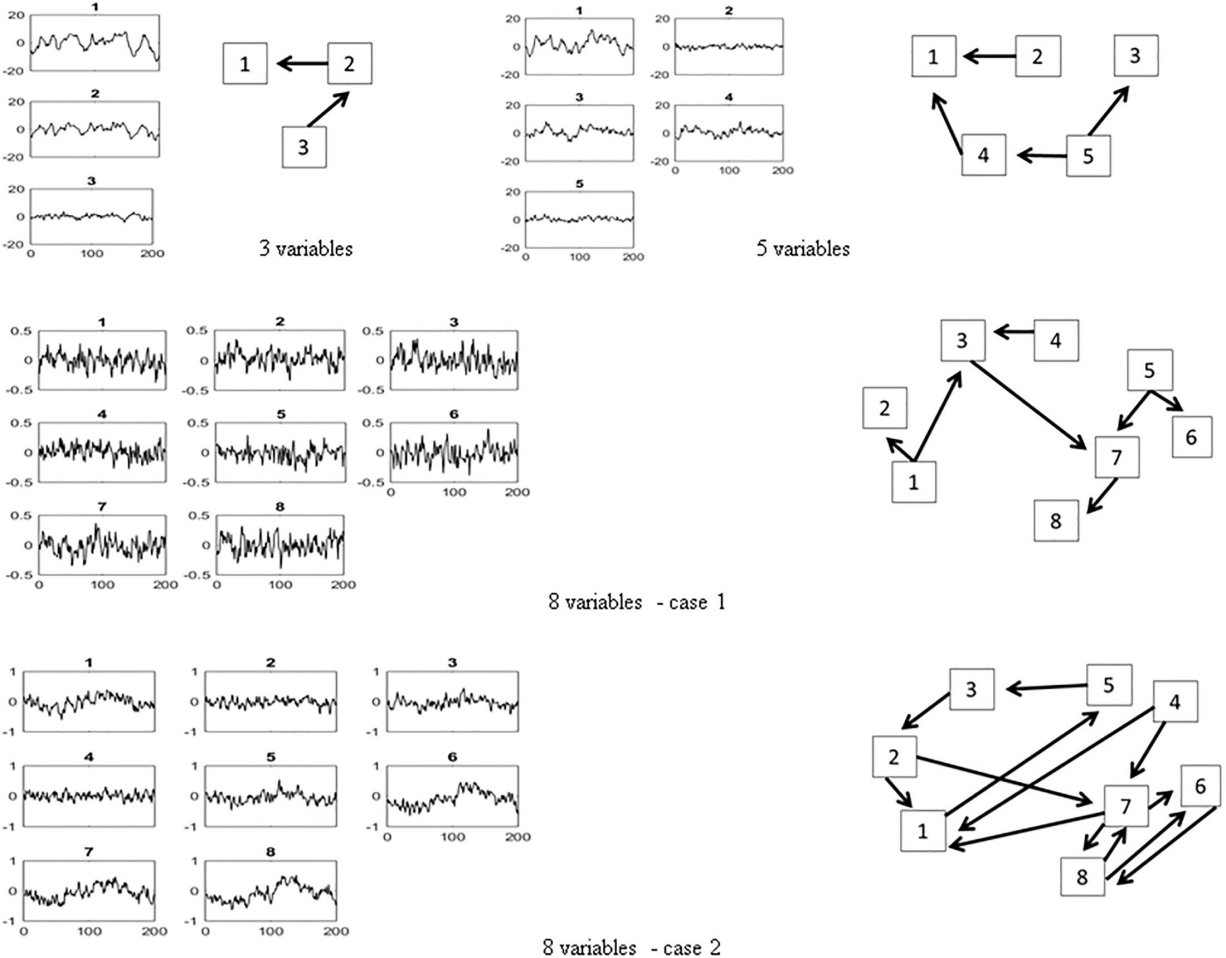
The 3, 5 and 8 variables simulation data. The 200 points data generated by the MAR(1) model are plotted on the Lhs, while the diagrams on Rhs represent the assumed relationships among the variables.

Blanks in the matrices represent zero elements. In Fig 2, the latter 200 points of generated data are plotted on the Left-hand side (Lhs) and the assumed causal relationships among variables are summarized for each case by diagrams on the Right-hand side (Rhs). The noise term is generated as a uniformly distributed random variable with zero mean and unit variance. Matlab has been used as a platform for data generation and numerical computations. The programs are collected as S1 Zip folder.

#### Study 1

We apply Granger causality analysis to the five-dimensional data, where the variables are given by

$$\begin{array}{l} x_{1}(t)=a_{11}(1)x_{1}(t-1)+\varepsilon_{11}(t)\;\text{and}\\ x_{1}(t)=a_{11}(1)x_{1}(t-1)+a_{12}(1)x_{2}(t-1)+\varepsilon_{12}(t). \end{array}$$

For Var(*ε*_11_) > Var(*ε*_12_), *x*_2_ causes *x*_1_. On the other hand, considering the following models:

$$\begin{array}{l} x_{2}(t)=a_{22}(1)x_{2}(t-1)+\varepsilon_{22}(t),\;\text{and}\\ x_{2}(t)=a_{21}(1)x_{1}(t-1)+a_{22}(1)x_{2}(t-1)+\varepsilon_{21}(t). \end{array}$$

If Var(*ε*_12_) > Var(*ε*_21_), *x*_1_ causes *x*_2_. The same procedures are perform for the pair *x*_1_ and *x*_3_, *x*_1_ and *x*_4_, …, and, *x*_4_ and *x*_5_. For comparison, we use the sum of residuals in a F-test, to calculate the F statistics. The computation is done using the *causality* function in the R package *library(vars)*. The derived p-values are given in the following matrices:

$$\left(\begin{array}{llll} p_{12} & p_{13} & p_{14} & p_{15}\\ p_{21} & \;p_{23} & p_{24} & p_{25}\\ p_{31} & \;p_{32} & \;p_{34} & p_{35}\\ p_{41} & p_{42} & p_{43} & p_{45}\\ p_{51} & p_{52} & p_{53} & p_{54} \end{array}\right)=\left(\begin{array}{llll} 2.0\times 10^{-4} & 1.93\times 10^{-6} & <2.2\times 10^{-16} & \text{1}.1\times 10^{-5}\\ 0.91 & \text{0}.11 & \text{0}.84 & \text{0}.97\\ \text{0}.35 & \text{0}.87 & \text{0}.040 & <2.2\times 10^{-6}\\ \text{0}.0053 & \text{0}.64 & \text{0}.28 & <2.2\times 10^{-16}\\ \text{0}.58 & \text{0}.96 & \text{0}.72 & \text{0}.69 \end{array}\right)$$

**Fig 3:**
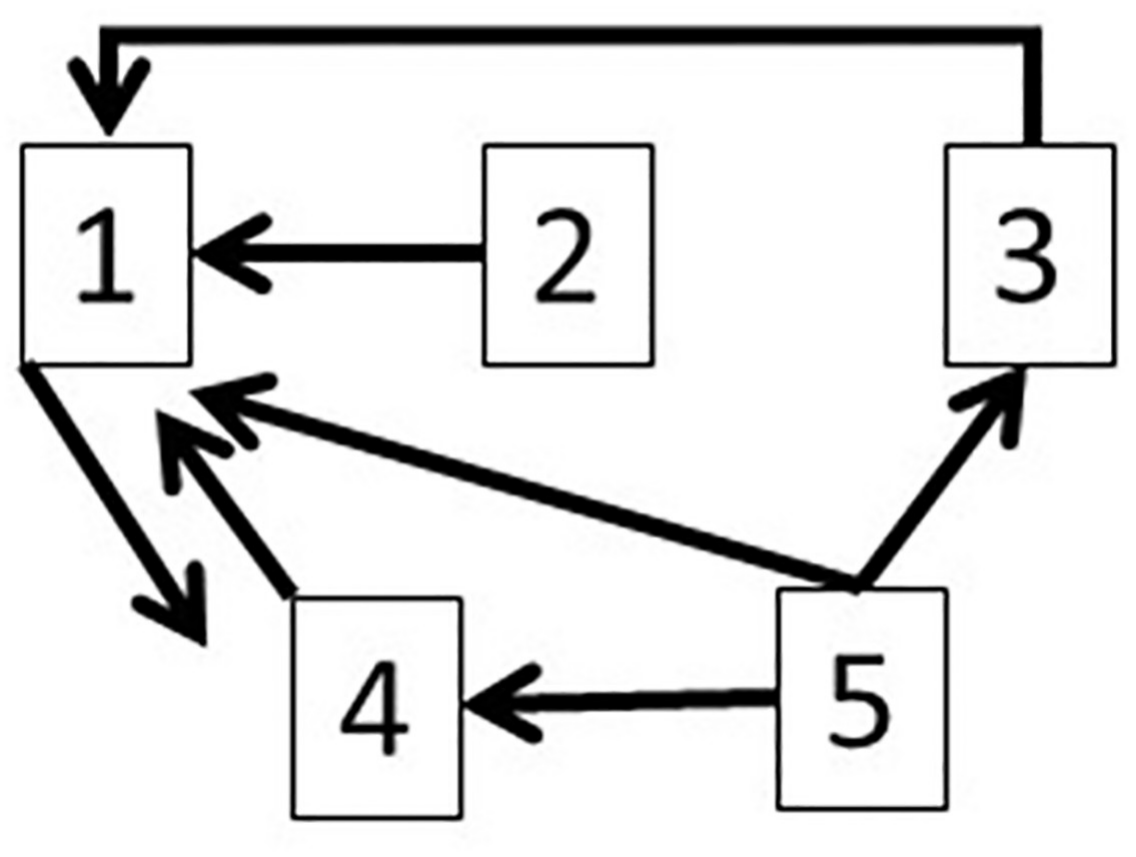
The inferred flows based on applying the Granger’s causality method to five-dimensional simulation data.

To avoid multiple testing problem, False Discovery Rate (FDR) [5] analysis was applied to the p-values. Finally, *p*_12_, *p*_13_, *p*_14_, *p*_15_, *p*_35_, *p*_45_, and *p*_41_ are selected at a 5% FDR significance level. The results are presented in Fig 3, which, in addition to the true flows, also captures three incorrect flows.

**Fig 4:**
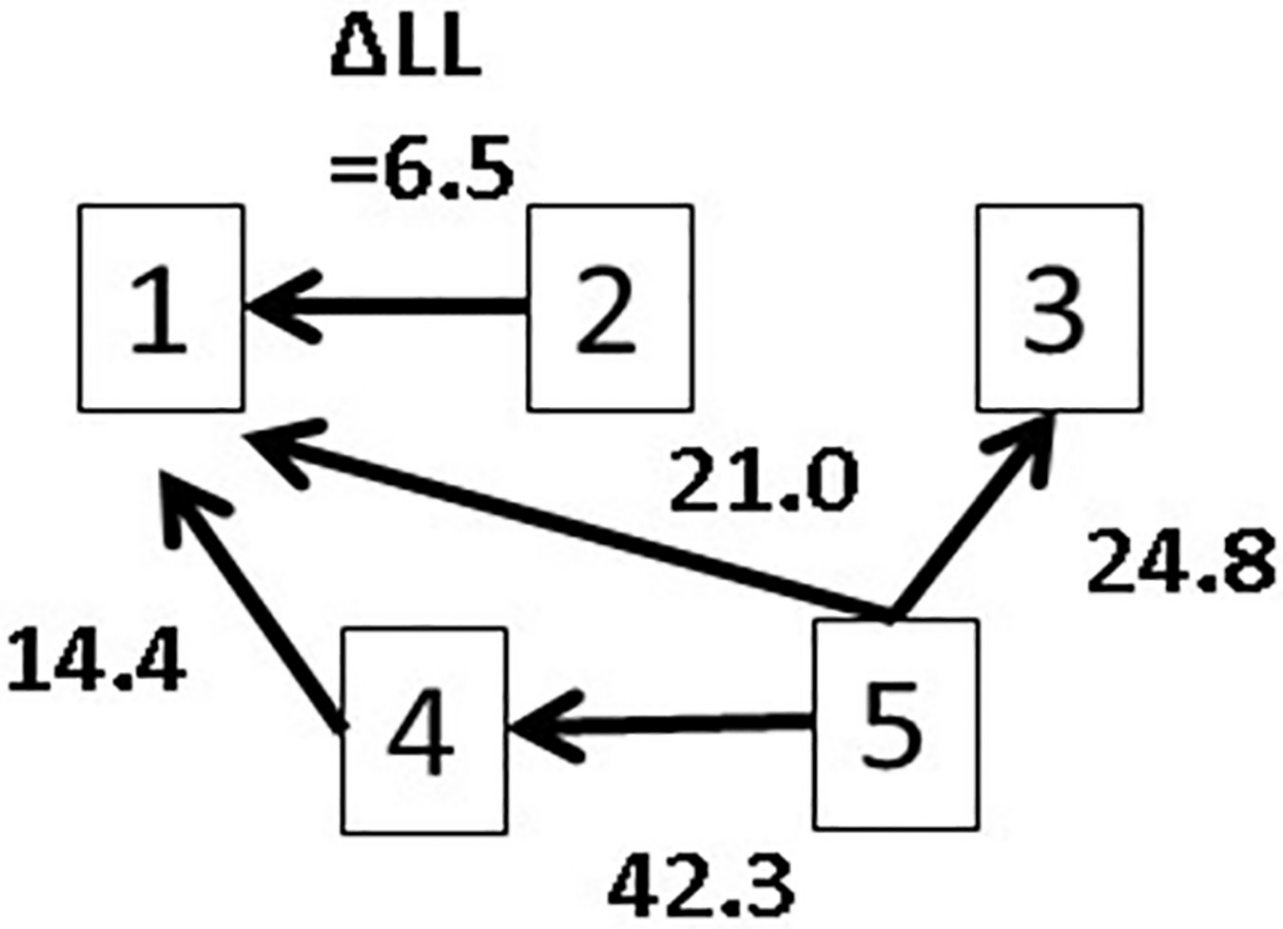
The calculated ΔLL values for five-dimensional simulation data and the inferred causal flow diagram.

Next, we applied the MAR model to the same dataset and calculated the ΔLL values. We summarize the results and inferred flows in Fig 4. This case captures only one extra flow in addition to the true flows. In fact, the extra flow is interpreted as a direct flow 5→ 1 that summarizes the flows 5→4→1. That is, the flows 5→ 1 and 5→4→1 follow an identical flow direction. Comparing the results of the MAR model to those using the bivariate model, we infer that using the former leads to more certain inference on causal flow direction, based on causal flow diagrams. We discuss how to detect direct/indirect flows in Study 2.

#### Study 2

We first investigate the sensitivity of the MAR model to data sample size by applying it respectively, to 50, 100, 150 and 200 points data for the various multidimensional variables. The values for Δ LL were then calculated using (4) , (5) and (10), based on the estimated coefficients by Levinson’s method [6] and the covariance matrix of the prediction error. The procedure was iterated 10,000 times and Table 1 summarizes the mean and standard deviation for number of correct/wrong flows for each case.

**Table 1:** Results for the sensitivity analysis. The table summarizes the mean (standard deviation) values for the number of correct/wrong flows, based on ΔLL > 2.5. The first row shows four variations of the simulation data. The figures in the parentheses are the assumed number of correct flows (relationships) among the variables. The first column represents the dimension of the simulation data**.**

	3var (2)		5var (4)		8var case1 (7)		8var case2 (13)	
size	correct	wrong	correct	wrong	correct	wrong	correct	wrong
50	1.8±0.37	1.4±0.98	3.9±0.26	4.5±2.35	6.9±0.33	47.6±5.7	12.8±0.59	41.0±5.76
100	2.0±0.21	0.8±0.70	4.0±0.09	1.9±1.08	6.5±0.67	4.4±2.01	11.8±1.09	6.2±2.46
150	2.0±0.10	0.7±0.57	4.0±0.00	1.2±0.56	6.8±0.46	2.0±1.34	12.3±0.83	4.2±1.95
200	2.0±0.10	0.6±0.54	4.0±0.00	1.1±0.45	6.9±0.31	1.1±1.02	12.6±0.7	3.2±1.52

**Fig 5:**
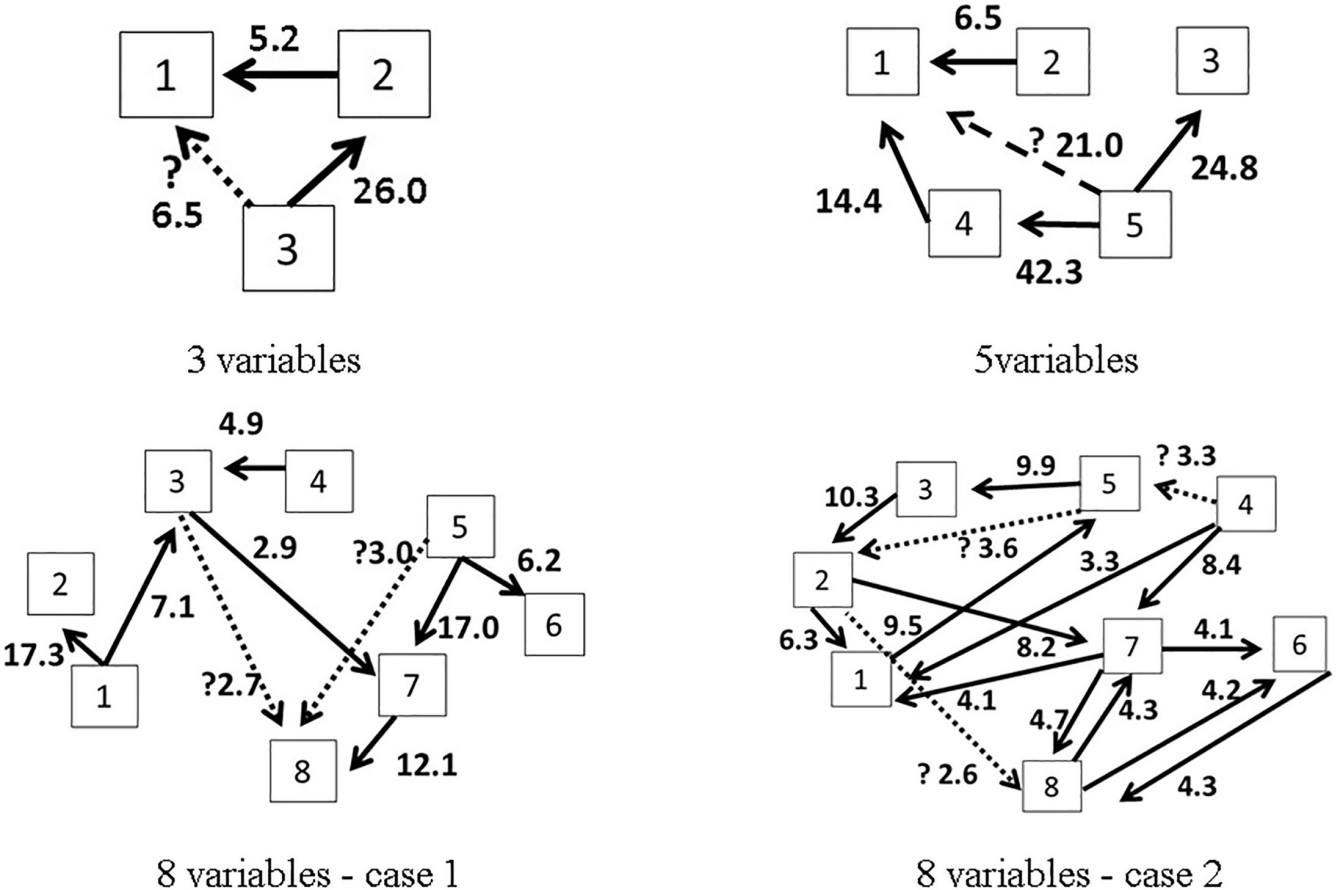
Estimated flows for data sample size of 200.

In general, the methodology appears to be accurate in capturing the correct number of flows (irrespective of the sample size), although number of incorrect flows appears to increase with increasing variable dimension. On the other hand, for 150–200-sample sizes, the correct number flows become stable, and indicate approximately the actual number of correct relationships. The number of wrong connections is mostly related to cases where intermediary flow relationships are ignored, i.e., when the flow A—> B—> C is registered as A—>C. We illustrate this in Fig 5, for a case involving a sample size of 200 data points.

The number beside each arrow is the ΔLL value that underpins our inference, where we have assumed the existence of a causal relationship when ΔLL > 2.5. In each diagram, the dotted line with the symbol ‘?’ represent flows that were not originally assumed in the model, but which evolved during the analysis as being significant (ΔLL>2.5). For example, in the case of 3 variables, the flow 3→1 was not originally assumed to exist in the model. However, this flow results direct connection for the two flows for 3→2 and 2→1. For the sake of consistency, we compare the models including only inferred influence by with the models including the flows drawn by the dotted lines. We prepare all possible combinations for the inferred influence by ΔLL as the alternative models. Furthermore, we applied the all possible models to the data and calculated the AIC again. The original model including only inferred influence by ΔLL are compared with the model indicating the minimum AIC among all possible models. The results are summarized in Table 2.

**Table 2:** Comparison of the models estimated by AIC.

variables	model	Log-likelihood	# parameters	AIC
3	Including inferred relationships $\left(\begin{array}{ccc} a_{11} & a_{12} & a_{13}\\ 0 & a_{22} & a_{23}\\ 0 & 0 & a_{33} \end{array}\right)$	-824.2	12	1672.4
	The best model: excluding 3→1 (*a*_13_)	-824.6	11	1671.3
5	Including inferred relationships $\left(\begin{array}{ccccc} a_{11} & a_{12} & 0 & a_{14} & a_{15}\\ 0 & a_{22} & 0 & 0 & 0\\ 0 & 0 & a_{33} & 0 & a_{35}\\ 0 & 0 & 0 & a_{44} & a_{45}\\ 0 & 0 & 0 & 0 & a_{55} \end{array}\right)$	-264.5	25	579.0
	The best model: excluding 5→1 (*a*_15_)	-265.4	24	528.8
8 case1	Including inferred relationships $\left(\begin{array}{cccccccc} a_{11} & & & & & & & \\ a_{21} & a_{22} & & & & & & \\ a_{31} & & a_{33} & a_{34} & & & & \\ & & & a_{44} & & & & \\ & & a_{53} & & a_{55} & & & \\ & & & & a_{65} & a_{66} & & \\ & & & & a_{75} & & a_{77} & \\ & & & & a_{85} & & a_{87} & a_{88} \end{array}\right)$	-408.6	53	923.3
	The best model: excluding 3→5 (*a*_53_) and 5→8 (*a*_*85*_)	-408.8	51	919.5
8 case2	Including inferred relationships $\left(\begin{array}{cccccccc} a_{11} & a_{12} & & a_{14} & & & a_{17} & \\ & a_{22} & a_{23} & & a_{25} & & & \\ & & a_{33} & & a_{35} & & & \\ & & & a_{44} & & & & \\ a_{51} & & & a_{54} & a_{55} & & & \\ & & & & & a_{66} & a_{67} & a_{68}\\ & a_{72} & & a_{74} & & & a_{77} & a_{78}\\ & a_{82} & & & & a_{86} & a_{87} & a_{88} \end{array}\right)$	-399.9	60	919.7
	The best model: excluding 5→2 (*a*_25_),4→5 (*a*_54_) and 2→8 (*a*_82_)	-398.3	57	910.6

The MAR model basically includes full relationships among all variables and the numerical algorithm outputs all coefficient estimates. The feedback relationships become complicated as the number of variables increases and the output for ΔLL > 2.5 leads to redundant connection including direct and undirect both flows as seen in the inferred relationships’ models. For all cases, the best model is a simple model without undirect flows. In real situations, the true relationships are usually unknown. Hence it is important that the results be evaluated with background knowledge about the data, especially for cases where the data sample size is small. Furthermore, more rigorous model fitting is required, and model selection must be guided by standard selection criteria as shown on Table 2.

### Causal analyses of Barents Sea capelin population dynamics

#### Data

**Fig 6:**
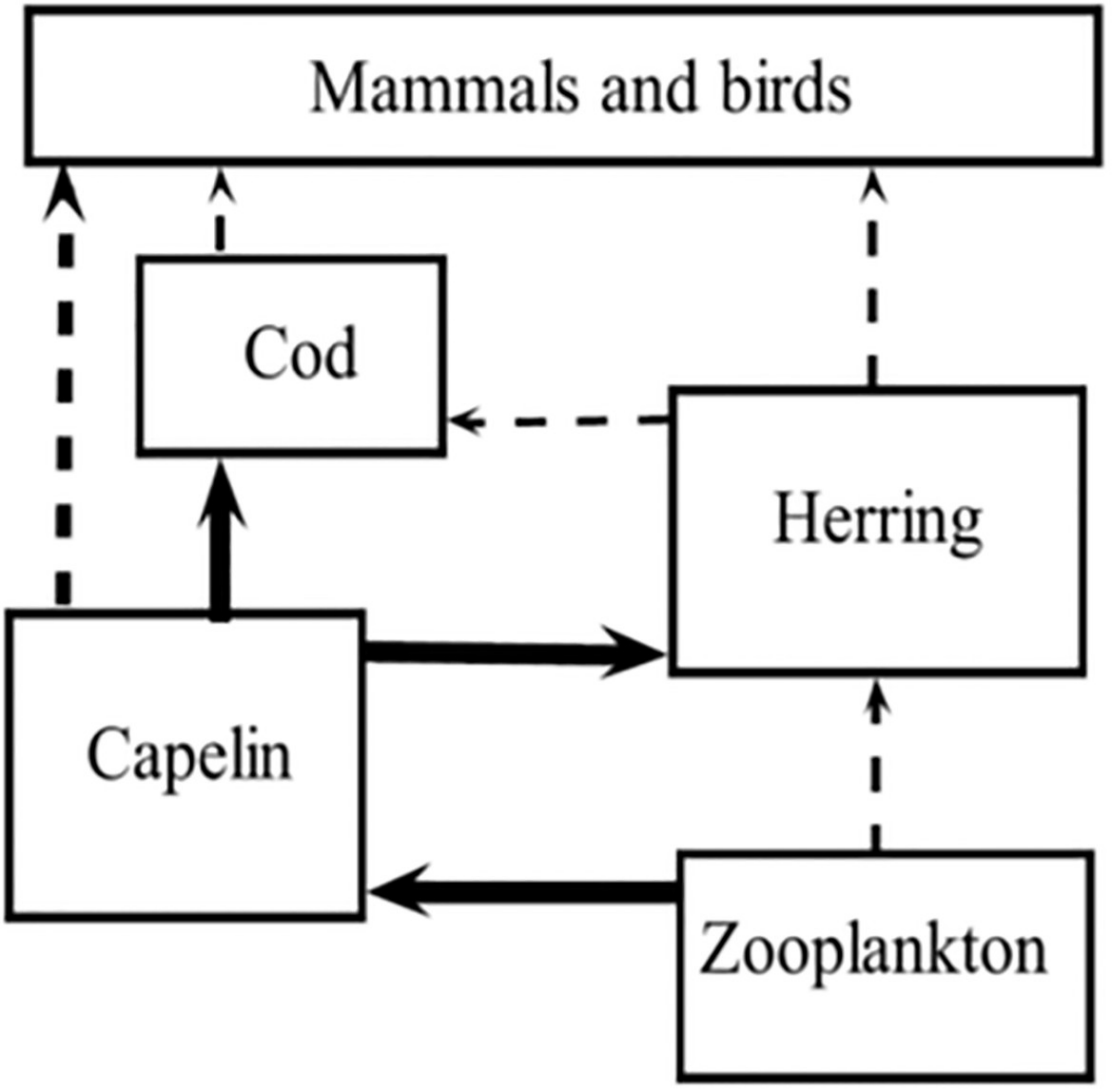
Food web of the Barents Sea. Showing capelin (focal species) and its link to both lower and higher trophic level species (redrawn after [22]).

**Fig 7:**
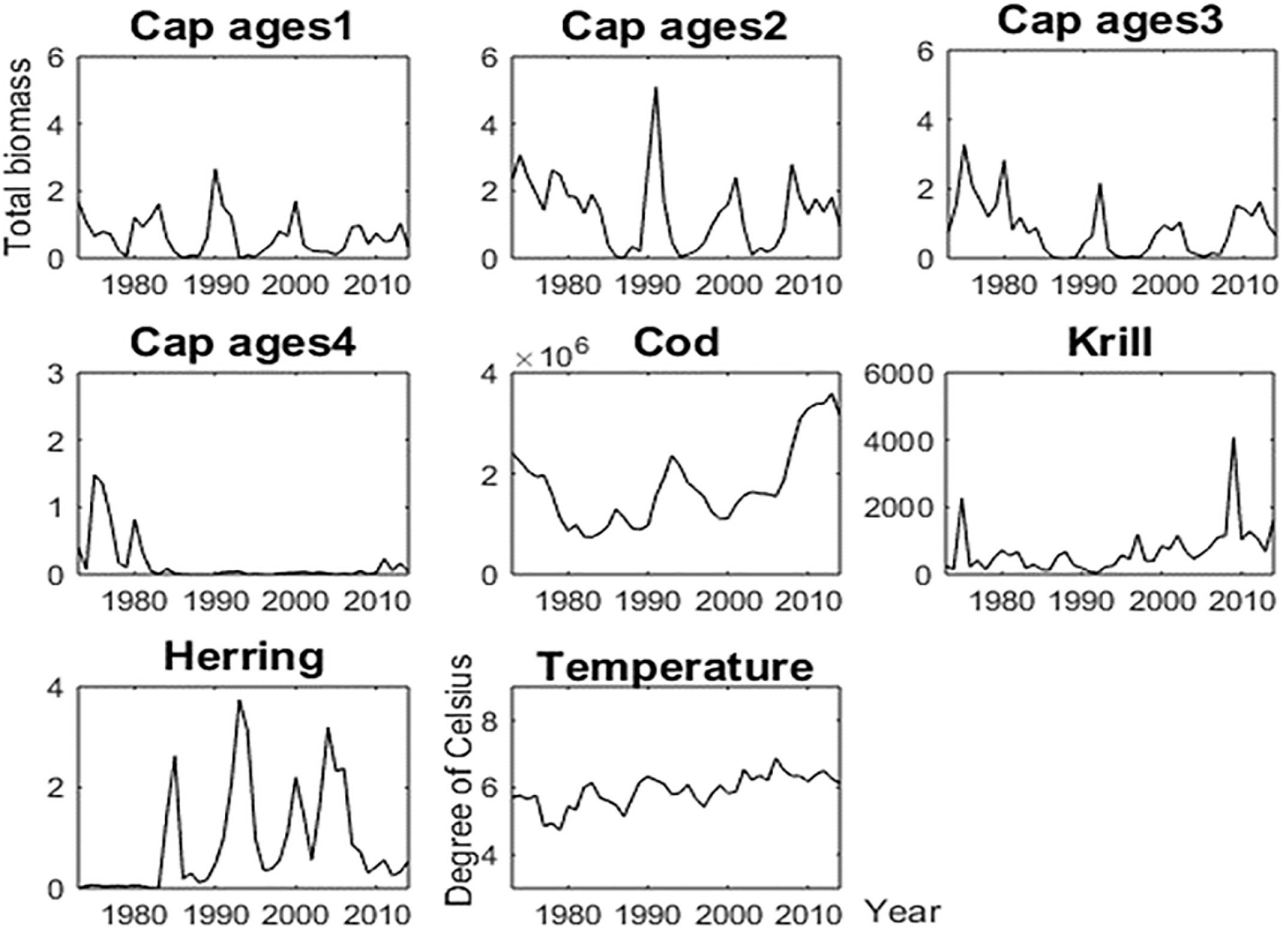
The five time series data for biotic and abiotic factors. The biotic and abiotic data including capelin ages 1–4 biomasses (×10^6^ kg on y-axis), cod and herring biomasses (kg on y-axis), krill density (×10^3^ *g* / *m*^2^ on y-axis), and Bird Island-Bear Island temperature (degree of Celsius). The x-axis indicates year.

Capelin is a short-lived (1–4 years) species that are the most important fish stock in the Barents Sea [18]. It is the main diet for Northeast Arctic cod and juvenile herring ([19], [20]). Several marine mammals, seabirds, kittiwakes and guillemots are also known to prey on capelin. The replenishment (recruitment) of the capelin stock is thought to be mainly regulated by the degree of juvenile herring predation on capelin larvae, and the predation by Northeast Arctic cod [21]. Both biotic (food supply—copepods, euphausiids, and hyperiids) and abiotic (ambient temperature) have been reported to affect capelin feeding, condition factor and distribution [22]. Fig 6 (re-drawn after Hjermann *et al*. [23]) represents a simplified food web of the Barents Sea, showing capelin (focal species) and its link to both lower and higher trophic level species. Based on Fig 6, we define the biotic dataset by the annual biomasses of capelin of ages 1–4, the total annual biomass of cod and herring, and the density of the krill biomass in the Barents Sea. The data are taken from the database of the WGIBAR [1], and for particularly for capelin, the survey procedure and biomass calculations can be found in Gjøsæter et al. [24]. We apply our methodology to infer causal relationships among the biotic observations, and the influence of temperature on the dynamics of the biotic data, i.e., evaluating the environmental effect on the four species. Fig 7 shows plots of the data used in the analyses.

Fig 7 shows the 5-dimensional time series matrix including capelin ages *, cod, krill, herring and temperature, where capelin* represents age-dependent capelin biomass at ages 1, 2, 3, or 4. We use capelin biomass data from 1973 to 2014, and associated covariate data within the same time range, where krill is used as a proxy for zooplankton in this paper.

### Results and discussion

#### Biotic data analyses

**Fig 8:**
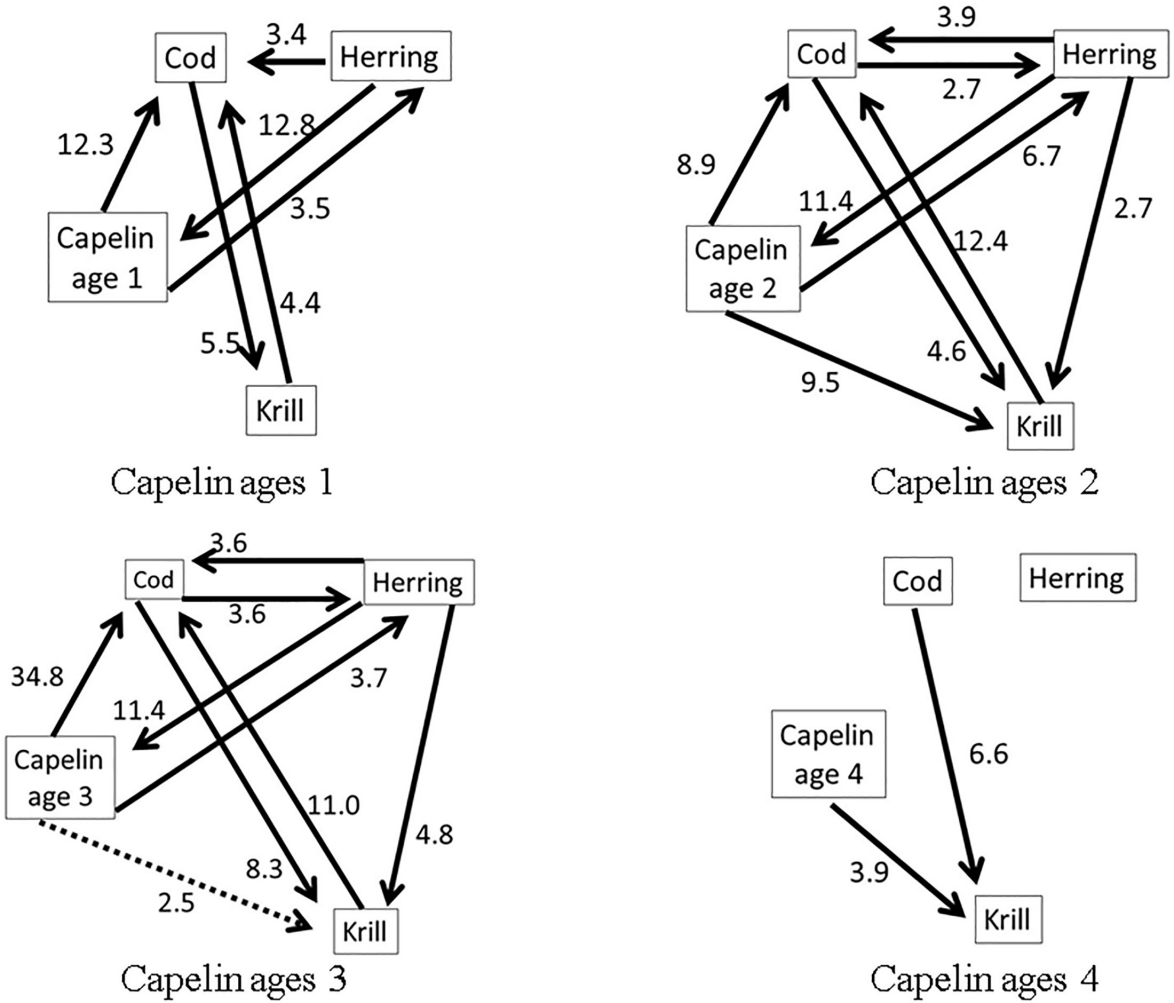
The inferred diagrams by ΔLL. The inferred flow diagrams for four species. The numeric value beside each arrow is indicative of the ΔLL value associated with flow direction.

We first applied the MAR model to the time series matrix, excluding temperature. The minimum AIC identified MAR (1) models for capelin ages 1 and 4, and MAR (2) models for capelin ages 2 and 3. Fig 8 presents the inferred flow diagrams based on ΔLL > 2.5.

From the diagrams, more causal relationships for capelin ages 2 and 3 appeared than causal relationships for age-1 capelin. The case for capelin ages 4 showed less interactions. Observation data for capelin ages 4 and above is usually sparse and unreliable (see [24]). Hence these age groups will be excluded from further discussions. The food web in Fig 6 indicated that cod and herring prey on capelin, while capelin and herring both prey on zooplankton. From our causal analyses, ages 1 to 3 capelin had influence on the variability of cod. Results for age-2 capelin indicated this age group is highly influenced by variability of the krill biomass. For age-3 capelin, we registered an influence from herring to krill that was absent in other capelin age groups. The influence level of herring on cod was slightly higher than that of cod on herring. The food web in Fig 6 also supports the weak relationship between zooplankton and herring, and between herring and cod.

**Fig 9:**
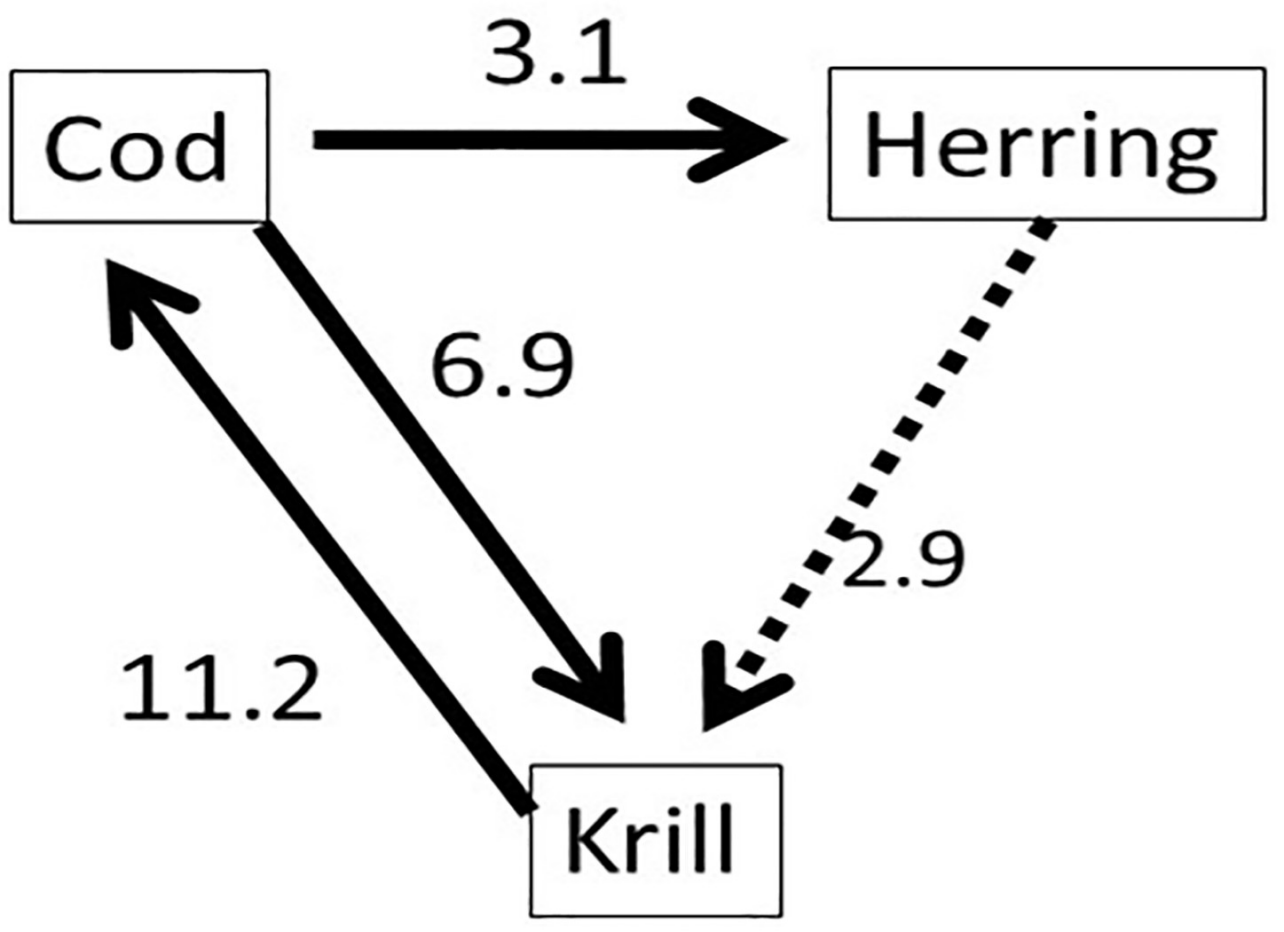
The ecosystem without capelin. The inferred flow diagrams.

From Fig 8, we notice that the causal relationship (existence and strength) between capelin and the other three species (cod, herring, krill) is different for the various capelin age groups. The dynamic between cod, herring and krill, in the absence of capelin, was analyzed, and the results are summarized in Fig 9. In this analysis, the feedback relationship between cod and krill appeared to be strong, while the relationships between cod and herring, and between krill and herring, appear to be less strong. This observation is supported by Bogstad et al. [25], who showed that ages 3–6 cod prey more on capelin than on krill. It can be inferred that capelin is the preferred prey for Northeast Arctic cod [19] and juvenile herring [20]. However, krill becomes important as prey for both species (cod and herring) in the absence of capelin. This is supported by the literature, especially diet compositions of cod during periods of capelin stock collapse [19].

#### The influence from temperature as an abiotic factor

**Fig 10:**
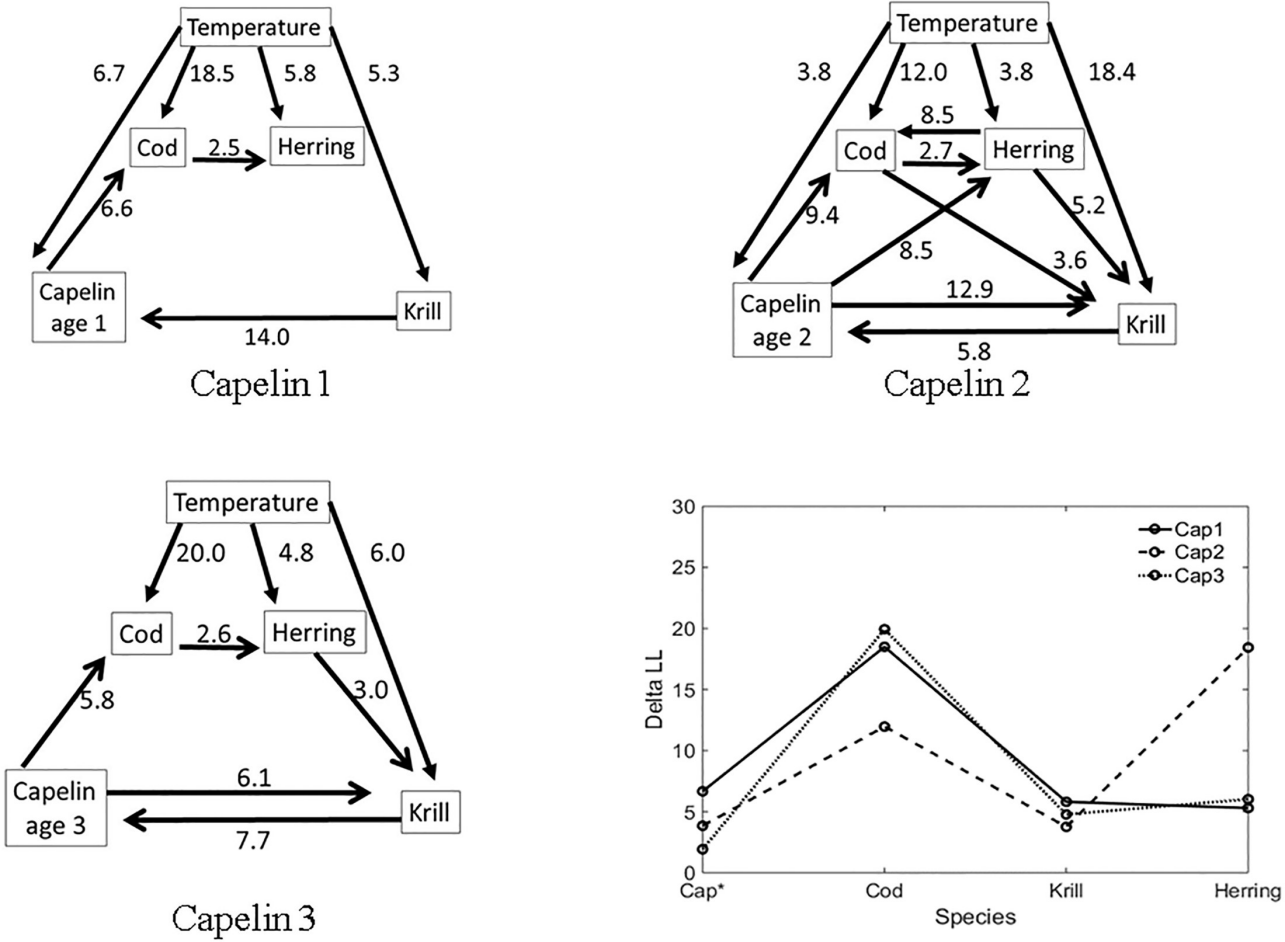
The inferred diagrams among four species and temperature, and the plots for the contributions from temperature to those species. In the plot, solid, dashed and dotted lines indicate the case for capelin age 1, 2 and 3, respectively.

As a further analysis, we applied the procedure for the biotic (four species) data to temperature, in order to investigate the influence of temperature on capelin of ages 1, 2 and 3. The identified regression orders were the same as previously; MAR (1) for capelin ages 1 and 3, and MAR(2) for ages-2 capelin. Since the influence from fish species to temperature is not realistic, elements in the AR coefficient matrix corresponding to this influence can be set to naught, i.e., *a*_51_ = *a*_52_ = *a*_53_ = *a*_54_ = 0, where *x*_1_, *x*_2_, *x*_3_, *x*_4_, and *x*_5_ represent the time series data for capelin, cod, krill, herring or temperature. For this case, the other coefficients may be estimated by any other methods rather than the Levinson’s algorithm. Applying numerical optimization procedures to estimate MAR coefficients sometime leads to unstable estimates that represent local minimum likelihood values. We therefore applied a full MAR model (including unrealistic relationships, such as the influence of fish on temperature) to the data and tested this against a model where unrealistic relationship has been excluded. The results of this test are summarized in Table 3. The AIC for the model without the influence of fish on temperature has consistently less AIC value than for the full MAR model. This applies for all capelin age groups considered. The inferred diagrams for 1–3 capelin ages and inference on the influence of temperature on the four-species considered was based on calculated ΔLL using the estimated MAR coefficients, as shown in Fig 10. Since the temperature was included as biotic factor to estimate MAR coefficients, the inferred diagrams indicate slightly different dynamics comparing with the inferred diagrams shown in Fig 8. The MAR orders for capelin age 2 and 3 were one while the orders in the case of Fig 8 were two. The relationship between capelin and herring were not captured in this case. Since MAR model includes full coefficients, the model that the abiotic factor is treated as an exogeneous variable seen in [26] could capture more significant relationship between capelin and herring. If we focus on the influence of temperature on the four-species, the obtained ΔLL was greater than 2.5 for all cases. The effect of temperature on cod was stronger (based on ΔLL) than on capelin and krill.

**Table 3:** Comparing full covariates model with the model excluding unrealistic relationships.

Capelin age	MAR model	Log-likelihood	# parameters	AIC
1	Full relationships	-247.7	25	545.3
	Excluding relationships fishes → temperature	-246.5	20	535.0
2	Full relationships	-253.8	50	607.5
	Excluding relationship fishes → temperature	-249.2	42	583.6
3	Full relationships	-235.6	25	520.6
	Excluding relationships fishes → temperature	-235.5	21	513.0

The unrealistic relationships mean the flow from biological factor to environmental factor such as fishes → temperature.

### Conclusions

We have provided a statistical methodology that integrates the pairwise causality methodology by Granger and Geweke, with the total causality approach defined by the Akaike’s power contribution. We have investigated the sensitivity of the method through simulation studies, using data generated by MAR models. For the simulation examples presented, our algorithm appears to be accurate in capturing the flow relationships. This is especially true for cases where the data sample size was at least 100, and the model parameter dimension did not exceed 8. We have also demonstrated the ability of the algorithm to capture redundancies in flow relationships. For the example with a sample size of 200, the algorithm captured both direct and intermediary (redundant) flows. A potential challenge with application of our methodology is the computational cost associated with larger data sets. The costs will be expected to increase with increasing number of possible flow combinations, and in the dimension of model parameters. A sequel paper will seek to address this computational drawback. The utility of the proposed methodology has been exemplified with real observations, by investigating the causal drivers of the Barents Sea capelin population dynamics. The sample size of the real observation is nearly 50, and the results are consistent with earlier studies on the trophic interactions between capelin, cod, herring and zooplankton in the Barents Sea. In addition, we have investigated the effect of temperature on four species using the MAR model. The results show the ability of our algorithm to identify observations that need to be treated as exogenous variables, and for which a MARX model (MAR model with exogenous variables) may be more appropriate.

A basic condition of our methodology is the assumption of data stationarity. For the examples we have considered, we have implicitly treated the data as stationary processes. This however, does not limit application of the methodology to non-stationary cases. When the data is nonstationary, we could first decompose it into non-stationary and stationary components, and then apply the computational procedure to the decomposed stationary part [27].

A caveat to our results is limitations imposed by the small sample size of the time series of real observations. The small sample size hinders an intuitive interpretation of the Akaike’s noise contribution in the short/middle/long individual frequency domain. This caveat notwithstanding, the proposed methodology gives us more in-depth understanding of the observations through integration of information along the entire frequency domain. It is a practical, first-step tool for analyzing the causal relationships in complex dynamical systems, such as, among marine populations and other biotic and abiotic ecosystem factors.

Finally, methodologies based on the Granger concept have been applied to complex ecosystems, as in recent articles [28] and [29]. The approach in [29] is a deterministic approach that does not consider stochastic properties the model. The results in [28] combines graph theory based on cross correlation and Granger causality. Since the method works for small sample sizes, a comparative study of our approach and that in [28] when applied to biotic and abiotic data will be given a further consideration.

## Supporting information

S1 Zip folder Matlab codes for generating simulation data and conducting the analysis. (ZIP)

## References

[1] Wiener N. The theory of prediction In Modern Mathematics for Engineers, ed. In: Beckenback EF editor. McGraw-Hill: New York:165–190: 1956.

[2] Glass L, Beuter A, and Larocque D. Time delays, oscillations, and chaos in physiological control systems, Math. Bios. 1988; 90:111–125.

[3] Francis T., Wolkovich E.M., Scheuerell M.D., Katz S.L., Holmes E.E., and Hampton S.E. Shifting regimes and changing interactions in the Lake Washington, U.S.A., plankton community from 1962–1994. PLOS ONE, 2014; 9; 2110363. 10.1371/journal.pone.0110363 PMC4206405 25338087

[4] Akaike H. On the use of a linear model for the identification of feedback systems, Annal. Inst. Stat. Math. 1968; 20: 425–439.

[5] Ozaki T. Time Series Modeling of Neuroscience Data. Chapman & Hall/CRC 2012.

[6] Akaike H.and Kitagawa G.edt. The Practice of Time Series Analysis: Springer: 1994.

[7] Baccalá LA and Sameshima K. Partial directed coherence: a new concept in neural structure determination. Biol. Cyber.2001; 84:463–474. 10.1007/PL00007990 11417058

[8] Bosch-Bayard J, Wong KFK, Okazaki S, Oshio R, Galka A, Ozaki T and Sadato N. Directed causality for non-stationary time series based on Akaike’s noise contribution ratio, FORMATH; 2012: 11: 121–131.

[9] Mann KH and Lazier JRN. Dynamics of marine ecosystems: biological-physical interactions in the oceans, Wiley-Blackwell; ISBN 978-1-4051-1118-8; 2005.

[10] Tett P, Gowen RJ, Painting SJ, Elliott M, Forster R, Mills DK, Bresnan E, Capuzzo E, Fernandes TF, Foden J, Geider RJ, Gilpin LC, Huxham M, Sathyendranath S, van der Molen J, Wilkinson M.Framework for understanding marine ecosystem health. Mar. Ecol. Prog. Ser. 2013; 494: 1–27.

[11] Cheung WWL, Watson R., and Pauly D. Signature of ocean warming in global fisheries catch. Nature; 2013: 497 7449:365–368. 10.1038/nature12156 23676754

[12] Johannesen E, Ingvaldsen RB, Bogstad B, Dalpadado P, Eriksen E, Gjøsæter H, Knutsen T, Skern-Mauritzen M, and Stiansen JE, Changes in Barents Sea ecosystem state, 1970–2009: climate fluctuations, human impact, and trophic interactions. ICES J. Mar. Sci. 2012; 69: 880–889.

[13] Tanokura Y and Kitagawa G. Power contribution analysis for multivariate time series with correlated noise sources, Adv. & Appl, in Stat. 2004; 4; 65–95.

[14] Granger CWJ. Investigating causal relations by econometric models and cross-spectral methods. Econ.1969; 37: 424–438.

[15] Geweke J. Measurement of linear dependence and feedback between multiple time series. J. Amer. Stat. Assoc.; 1982: 77:304–313.

[16] Kolmogorov AN. Stationary sequences in Hilbert space, Bull. Math. Univ. Moscou 1941; 5:3–14.

[17] Akaike H. A new look at statistical model identification, IEEE Tran. Automat. Contrl. 1974; AC-19: 716–723.

[18] Gjøsæter H and Ushakov NG. Capelin in the Barents Sea. In Proceedings of the 10^th^ Norwegian-Russian Symposium. In: Bjordal Å, Gjøsæter H and Mehl S editors. Bergen. 2003; 8–17: H.

[19] Gjøsæter H, Bogstad B, and Tjelmeland S. Ecosystem effects of the three capelin stock collapses in the Barents Sea. Mar. Biol. Res.2009; 5: 40–53.

[20] Hallfredsson EH and Pedersen T. Effects of predation from juvenile herring *(clupea harengus)* on mortality rates of capelin *(mallotus villosus)* larvae. Canad. J. Fish. Aqua. Sci.2009; 66:1693–1706.

[21] Gjøsæter H, Hallfredsson EH, Mikkelsen N, Bogstad B, and Pedersen T. Predation on early life stages is decisive for year-class strength in the Barents Sea capelin *(Mallotus villosus)* stock. ICES J. Mar. Sci. 2015; p. fsv177.

[22] Orlova EL, Boitsov AD, Dolgov AV, Rudneva GB, and Nesterova VN. The relationship between plankton, capelin, and cod under different temperature conditions. ICES Journal of Marine Science. 2004; 62; 1281–1292.

[23] Hjermann DØ, Stenseth NC, and Ottersen G, Indirect climate forcing of the Barents Sea capelin: a cohort effect. Mar. Ecol. Prog. Ser. 2004; 273: 229–238.

[24] Gjøsæter H, Bogstad B, and Tjelmeland S, Assessment methodology for Barents Sea capelin, *Mallotus villosus (Muller)*. ICES J. Mar. Sci; 2002: 59:1086–1095.

[25] Bogstad B, Gjøsæter H, Haug T, and Lindstrøm U. A review of the battle for food in the Barengs Sea: cod vs. marine mammals. Front. Ecol. Evol.; 2015: 25: 10.3389/fevo.2015.00029

[26] Hampton S.E., Scheuerell M.D. and Schindler D.E. Coalescence in the Lake Washington story: Interaction strengths in a planktonic food web. Limnol. Oceanogr., 2006; 51;2042–2051.

[27] Kato H., Naniwa S., and Ishiguro M., A Bayesian multivariate nonstationary time series model for estimating mutual relationships among variables, Journal of Econometrics, 1996; 75; 147–161.

[28] Damos P. Using multivariate cross correlations, Granger causality and graphical models to quantify spatiotemporal synchronization and causality between pest populations. BMC Ecol, 2016; 16:33; 10.1186/s12898-016-0087-7 27495149 PMC4974811

[29] Sugihara G., May R., Ye H., Hsieh C., Deyle E., Fogarty M. and Munch S. Detecting causality in complex ecosystems. Science 2012; 338. 10.1126/science.1227079 22997134

