# Correction: An improved methodology for quantifying causality in complex ecological systems

**DOI:** 10.1371/journal.pone.0217195

**Published:** 2019-06-11

**Authors:** Hiroko Kato Solvang, Sam Subbey

The second sentence in the Introduction should have cited reference 14 instead of 2. The correct sentence should read: Granger [14] followed this notion of causality, and applied it to the analysis of economic time series data.

14. Granger CWJ. Investigating causal relations by econometric models and cross-spectral methods. Econ.1969; 37: 424–438.

The fifth sentence in the Introduction should have cited reference 15 instead of 3. The correct sentence should read: Geweke [15] expanded on Granger’s idea to define a measure for model comparison based on using the quasi-likelihood function.

15. Geweke J. Measurement of linear dependence and feedback between multiple time series. J. Amer. Stat. Assoc.; 1982: 77:304–313.

The seventh sentence in the Introduction should have cited reference 4 instead of 5. The correct sentence should read: Here, we define feedback as being present when given bivariate time series, each of them is mutually causal to the other [4].

4. Akaike H. On the use of a linear model for the identification of feedback systems, Annal. Inst. Stat. Math. 1968; 20: 425–439.

A reference is omitted from the penultimate sentence of the first paragraph in the Introduction. The correct sentence should read: The methodology is referred to as the Akaike’s total causality approach [5].

5. Ozaki T. Time Series Modeling of Neuroscience Data. Chapman & Hall/CRC. 2012.

The final sentence beneath the “multivariate auto-regressive model and model selection” subheading in the Methods should have cited reference 17 instead of 2. The correct sentence should read: The AR order is identified by statistical model selection approaches, such as, the Akaike Information Criterion (AIC) [17], defined by

AIC=−2×log−likelihood+2×#model's parameters. (2)

17. Akaike H. A new look at statistical model identification, IEEE Tran. Automat. Contrl. 1974; AC-19: 716–723.

The reference in eighth sentence beneath the “Study 1” subheading of the Simulation study section is incorrect. The correct sentence should read: To avoid multiple testing problem, False Discovery Rate (FDR) (Benjamini and Hochberge, 1995) analysis was applied to the p-values.

The correct citation is: Benjamini, Y. and Hochberge, Y. 1995 Controlling the false discovery rate: a practical and powerful approach to multiple testing, J.R.Statistics, Soc.B, 57:289–300.

Beneath the “Data” subheading of the Causal analyses of Barents Sea capelin population dynamics section, reference 1 is mistakenly cited in the first half of the eight sentence. The correct sentence should read: The data are taken from the database of the WGIBAR (ICES, 2016), and for particularly for capelin, the survey procedure and biomass calculations can be found in Gjøsæter et al. [24].

The correct citation is: ICES. 2016 Final report of the working group on the integrated assessment of the Barents Sea (WGIBAR). ICES CM, 2016/SSGIEA:04:123 p.

The Fig 6 caption should have cited reference 23 instead of 22. Please see the complete, correct Fig 6 caption and image here.

**Fig 6:**
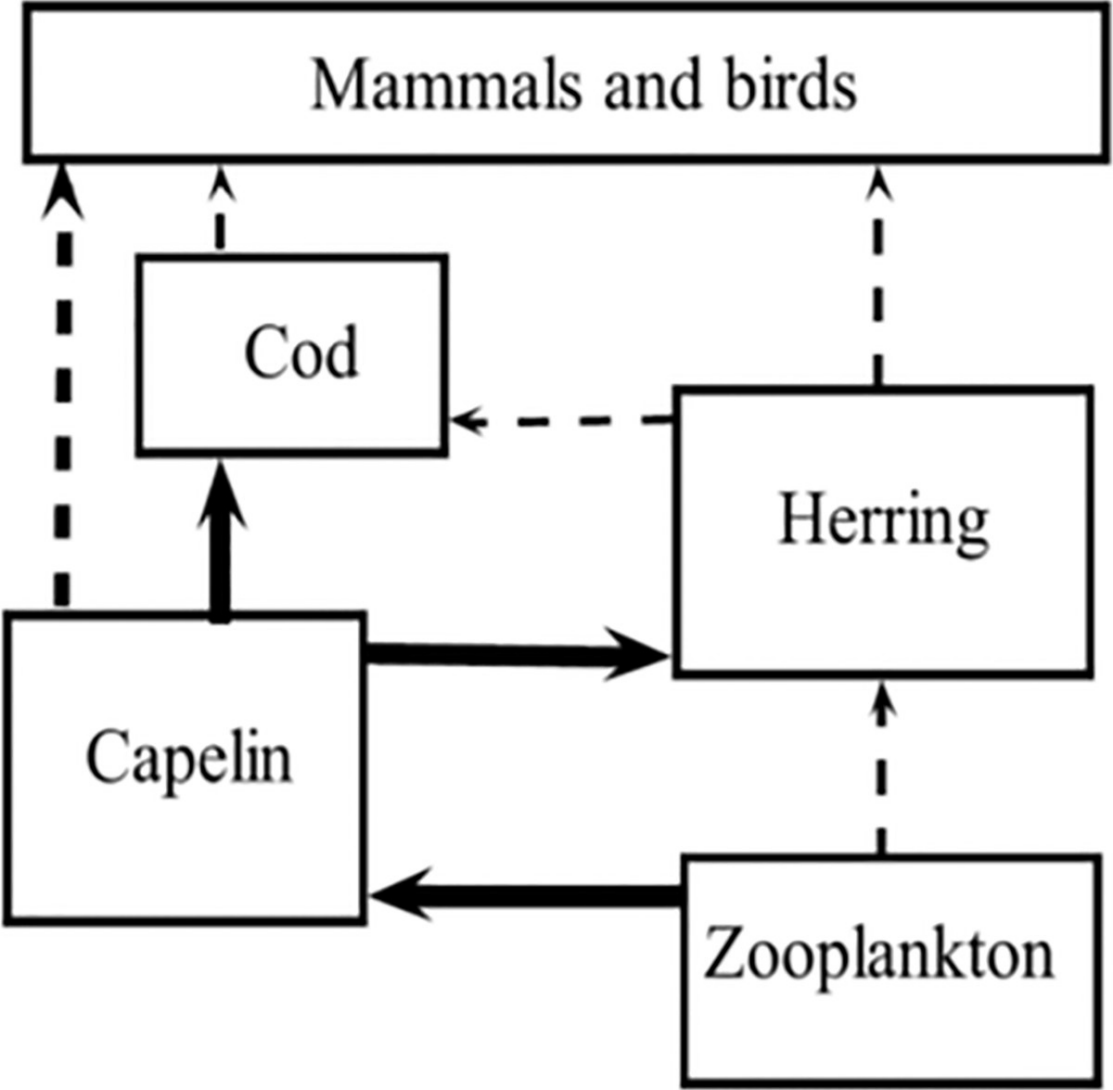
Food web of the Barents Sea. Showing capelin (focal species) and its link to both lower and higher trophic level species (redrawn after [23]).

23. Hjermann DØ, Stenseth NC, and Ottersen G, Indirect climate forcing of the Barents Sea capelin: a cohort effect. Mar. Ecol. Prog. Ser. 2004; 273: 229–238.

Beneath the “The influence from temperature as an abiotic factor subheading” in the Results and Discussion, reference 1 is incorrectly omitted from the fourteenth sentence. The correct sentence should read: Since MAR model includes full coefficients, the model that the abiotic factor is treated as an exogeneous variable seen in [3, 26] could capture more significant relationship between capelin and herring.

3. Francis T., Wolkovich E.M., Scheuerell M.D., Katz S.L., Holmes E.E., and Hampton S.E. Shifting regimes and changing interactions in the Lake Washington, U.S.A., plankton community from 1962–1994. PLOS ONE, 2014; 9; 2110363.
